# Navigating the ethical landscape of artificial intelligence in radiography: a cross-sectional study of radiographers’ perspectives

**DOI:** 10.1186/s12910-024-01052-w

**Published:** 2024-05-11

**Authors:** Faten Mane Aldhafeeri

**Affiliations:** Collage of Applied Medical Sciences, University of Hafr Albatin, P.O.Box 31991, Hafr Albatin, Saudi Arabia

**Keywords:** Artificial intelligence, Radiography, Diagnosis

## Abstract

**Background:**

The integration of artificial intelligence (AI) in radiography presents transformative opportunities for diagnostic imaging and introduces complex ethical considerations. The aim of this cross-sectional study was to explore radiographers’ perspectives on the ethical implications of AI in their field and identify key concerns and potential strategies for addressing them.

**Methods:**

A structured questionnaire was distributed to a diverse group of radiographers in Saudi Arabia. The questionnaire included items on ethical concerns related to AI, the perceived impact on clinical practice, and suggestions for ethical AI integration in radiography. The data were analyzed using quantitative and qualitative methods to capture a broad range of perspectives.

**Results:**

Three hundred eighty-eight radiographers responded and had varying levels of experience and specializations. Most (44.8%) participants were unfamiliar with the integration of AI into radiography. Approximately 32.9% of radiographers expressed uncertainty regarding the importance of transparency and explanatory capabilities in the AI systems used in radiology. Many (36.9%) participants indicated that they believed that AI systems used in radiology should be transparent and provide justifications for their decision-making procedures. A significant preponderance (44%) of respondents agreed that implementing AI in radiology may increase ethical dilemmas. However, 27.8%expressed uncertainty in recognizing and understanding the potential ethical issues that could arise from integrating AI in radiology. Of the respondents, 41.5% stated that the use of AI in radiology required establishing specific ethical guidelines. However, a significant percentage (28.9%) expressed the opposite opinion, arguing that utilizing AI in radiology does not require adherence to ethical standards. In contrast to the 46.6% of respondents voicing concerns about patient privacy over AI implementation, 41.5% of respondents did not have any such apprehensions.

**Conclusions:**

This study revealed a complex ethical landscape in the integration of AI in radiography, characterized by enthusiasm and apprehension among professionals. It underscores the necessity for ethical frameworks, education, and policy development to guide the implementation of AI in radiography. These findings contribute to the ongoing discourse on AI in medical imaging and provide insights that can inform policymakers, educators, and practitioners in navigating the ethical challenges of AI adoption in healthcare.

**Supplementary Information:**

The online version contains supplementary material available at 10.1186/s12910-024-01052-w.

## Background

The rapid integration of artificial intelligence (AI) into the field of radiography has ushered in a new era of diagnostic capabilities, workflow efficiency, and decision support. AI technologies have become increasingly prevalent in medical imaging; therefore, navigating the ethical landscape surrounding their implementation is imperative [1, 2].

The introduction of AI in radiography brings forth a myriad of potential benefits, including improved diagnostic accuracy, enhanced workflow efficiency, and capability of handling vast amounts of imaging data. However, these advances pose ethical challenges that require careful examination. Radiographers have a pivotal role in the successful integration of AI technologies as integral members of the healthcare team responsible for acquiring and interpreting medical images. Understanding their perspectives on the ethical implications of AI is crucial for fostering the responsible and patient-centered use of these technologies [1, 2].

Ethical considerations surrounding the integration of AI into the field of radiography have received significant attention and have become a topic of extensive discourse among researchers and professionals. The study of ethical considerations in AI is progressing with advancements and integration into the field of imaging. This conflict emphasizes the importance of incorporating ethical principles into radiography when utilizing AI technology in radiography [3]. The ethical considerations in the adoption of AI in radiography mirror those in broader healthcare AI applications but are uniquely contextualized within the realm of medical imaging. For instance, patient confidentiality is a critical concern because AI algorithms process and analyze sensitive medical images [4]. The potential for bias in algorithms, if not carefully addressed, can lead to disparities in diagnostic outcomes, impact patient care, and exacerbate existing healthcare disparities. Furthermore, issues regarding accountability and transparency have arisen because AI systems often operate as complex “black boxes,” thereby making understanding their decision-making processes challenging [5, 6]. Problematic judgments reflecting biases in training data have previously been demonstrated in AI systems applied in nonmedical domains. For example, software meant to assist judges in determining a defendant’s sentence by estimating the likelihood of recidivism has a disturbing evidence of bias. In the absence of genetic research in particular groups, an algorithm developed to forecast outcomes based on genetic data may similarly exhibit bias [4, 7].Despite the growing importance of AI in radiography, few studies have explored the ethical dimensions of its implementation from the perspective of radiographers. Previous studies [8, 9] have primarily focused on the technical aspects and diagnostic performance of AI systems, thereby leaving a critical gap in understanding how professionals perceive and navigate these technologies ethically. Therefore, the aim of this study was to fill this gap by conducting a cross-sectional investigation of radiographers’ perspectives on the ethical landscape of AI in radiography. By gaining insight into their views, concerns, and experiences, we seek to inform the development of ethical guidelines, educational programs, and policies that promote the responsible integration of AI in radiographic practice.

## Methods

### Survey design

The study has been approved by the Local Research Ethics Committee of Health Affairs, Hafr Albatin (1H00014532/S). This study employed a cross-sectional research design to collect data at a single point in time, allowing for a snapshot of radiographers’ perspectives on the ethical landscape of AI in radiography. The use of a cross-sectional approach enables the exploration of diverse viewpoints and identification of trends among radiographers.

A survey was used to investigate the ethical challenges radiographers face when incorporating AI technologies into their routine radiography practices. The survey comprised three distinct sections. The first section gathered demographic information such as age, sex, qualifications, work experience, and occupation. The second section was used to assess participants’ knowledge of AI and its applications in radiology, focusing on their familiarity with current AI applications. The third section was used to examine the impact of ethical considerations on radiographers’ practices, particularly concerning the influence of AI on their professional autonomy and decision-making authority.

The survey instrument used in this study was developed to collect relevant data. Before distribution to the participants, the survey underwent a comprehensive pilot and testing phase to ensure its validity and reliability. Feedback from a group of radiographers and faculty members (which included radiographers and radiologists) who were not involved in the primary investigation was incorporated to enhance and refine the survey instrument. See supplementary material.

### Participants

This study used a convenience sample of radiographers practicing in various healthcare settings. The inclusion criteria were licensed radiographers with a minimum of 1 year of professional experience. Radiographers with expertise in AI or those actively using AI technologies in their practice were encouraged to participate to ensure a diverse range of perspectives.

Recruitment efforts involved collaboration with radiography departments in healthcare institutions. Invitations to participate, including a brief explanation of the purpose of the study and a link to the online questionnaire, were distributed through social media groups. Participation was voluntary and informed consent was obtained from each participant.

### Data collection

The survey was conducted through an online platform using the Google Forms web-based application developed by Google Inc. (Mountain View, CA, USA). Before commencing the survey, participants were required to provide informed consent and ensure that their participation in the study was voluntary. In addition, to maintain anonymity, the survey application incorporated an anonymous response feature, allowing respondents to provide their input without revealing their personal information. The survey commenced on August 2, 2023, and remained accessible for the duration of 12 weeks. The link was disseminated via email to the contacts and promoted on various social media platforms.

Quantitative section: Participants responded to closed-ended questions on demographics, familiarity with AI in radiography, and the perceived importance of various ethical considerations related to AI use. Likert scales were used to measure the participants’ attitudes and perceptions.

Qualitative section: Open-ended questions were included to gather in-depth insights into the radiographers’ experiences and concerns regarding the ethical aspects of AI in radiography. The participants were encouraged to provide detailed responses to capture the richness of their perspectives.

### Data analysis

Data were imported into Statistical Package for Social Sciences Version 22 (IBM, Inc., Armonk, NY, USA). The Likert scale responses were transformed into a continuous data format by allocating specific values, which were as follows: “strongly disagree” was assigned a value of “1,” “disagree” was assigned a value of “2,” “neutral” was assigned a value of “3,” “agree” was assigned a value of “4,” and “strongly agree” was assigned a value of “5.” The application of reverse scoring was contingent on the formulation of the questions. The attainment of elevated scores was suggestive of individuals harboring a favorable perspective toward the ethical considerations of AI in the field of radiography, whereas lower scores were suggestive of individuals holding an unfavorable viewpoint.

### Rigor and trustworthiness

To enhance the rigor of the study, steps were taken to ensure data validity and reliability. Piloting the questionnaire with a small group of radiographers helped to identify and address any ambiguities or potential issues. Additionally, the use of established qualitative research methodologies such as intercoder reliability checks contributed to the trustworthiness of the thematic analysis.

## Results

### Demographic characteristics of the participants

The study included 388 respondents of whom 269 (72.2%) respondents were men and 119 (27.8%) respondents were women. Most participants were 33–43 years old. The number of years of experience as radiographers was 17 ± 4 years (expressed as the average ± standard deviation) with a range of 5–25 years. Additional demographic details are presented in Table 1.

**Table 1:** Demographic characteristics of the participants

Demographic variable	Frequency (*n*)	Percentage (%)
Age (y)		
22–32 33–43 44–54 > 54	117 190 81 0	30.15 49.96 20.8 0
Sex		
Male Female	269 119	72.2 27.8
Years of experience		
Mean (± standard deviation)	17 (± 11.8)	
< 5 y 5–9 y 15–14y 15–19 y > 20 y	25 37 116 174 36	6.4 9.53 29.9 44.8 9.3
Highest level of education		
Diploma Bachelor’s degree Master’s degree PhD	128 231 25 4	33 59.5 6.4 1

### Familiarity with AI in radiography

A substantial number (44.8%) of the participants reported that they were unfamiliar with the integration of AI in radiography, whereas 32.4% of participants indicated a moderate level of familiarity. A small proportion (22.8%) reported being familiar or very familiar with AI in radiography.

### Ethical considerations

Participants were asked to rate the importance of various ethical considerations related to AI in radiography on a Likert scale ranging from 1 (“extremely not important/not willing at all, very unconcerned”) to 5 (“extremely important, very willing, and very concerned”). Most radiographers (32.9%) expressed uncertainty about the significance of transparent AI systems in radiology and offered explanations for their decisions. The most frequently chosen answer among the respondents was “neutral.” By contrast, a significant proportion (36.9%) of respondents expressed the belief that AI systems utilized in radiology should be transparent and offer explanations for their decision-making processes. Approximately, 27.8% of respondents exhibited a degree of uncertainty in acknowledging and understanding the potential ethical issues that may arise from integrating AI in radiology. This uncertainty was reflected by the respondents’ selection of the “neutral” response. A significant preponderance (44%) of respondents expressed agreement regarding the potential increase in ethical dilemmas associated with the implementation of AI in radiology. However, that this viewpoint represents a prevailing sentiment among the surveyed population is important to note.

A significant number (41.5%) of participants stated that the implementation of dedicated ethical guidelines is necessary for utilizing AI in radiology. However, a substantial proportion (28.9%) of participants held the opposing perspective and suggested that ethical guidelines are not required for the integration of AI in radiology. A notable proportion (29.9%) of the participants expressed uncertainty regarding the significance of ethical guidelines pertaining to AI in radiology.

**Fig. 1:**
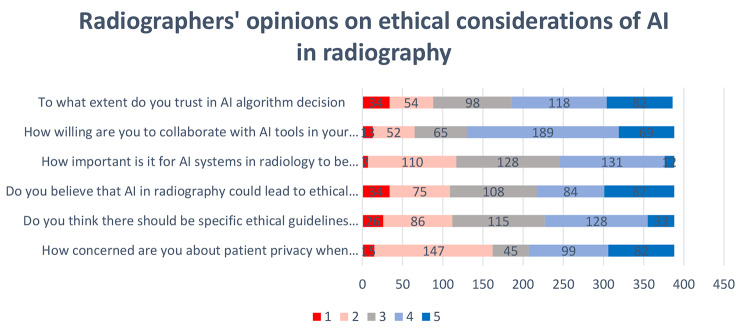
Likert scale bar chart results. The participants’ views regarding the ethical considerations of utilizing artificial intelligence (AI) in radiography are summarized

In light of growing concerns surrounding patient privacy in the context of utilizing AI in radiology, the respondents expressed their perspectives on this matter. A significant proportion (46.6%) of respondents expressed concerns about patient privacy. However, a slightly smaller percentage (41.5%) of respondents did not share the same level of concern (Fig. 1.).

**Fig. 2:**
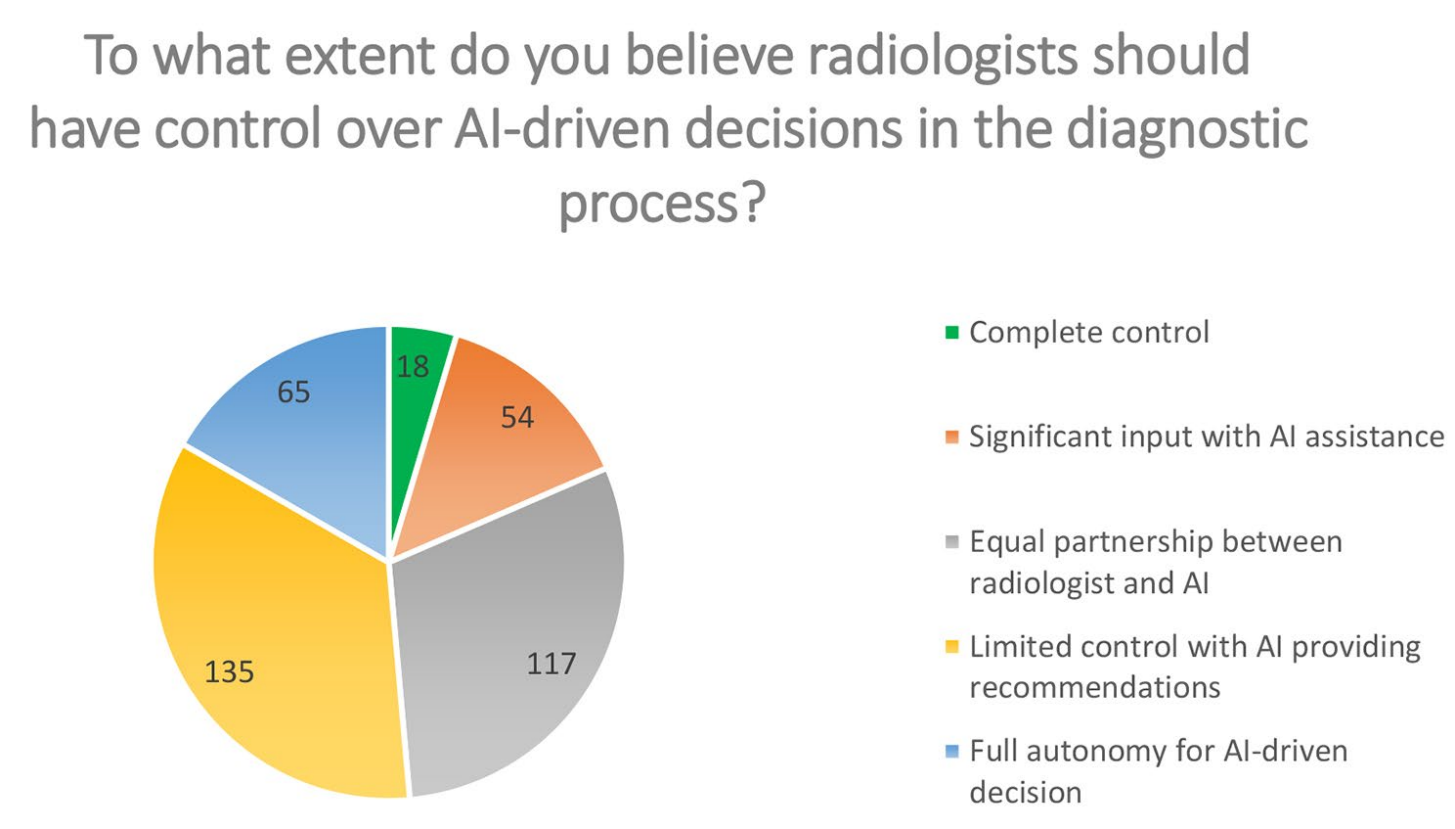
Pie chart results. The participants*’* views regarding radiologists*’* role in artificial intelligence (AI)-driven decisions in the diagnostic process are summarized

In relation to a statement about the level of control that radiologists should have in AI-driven decisions during the diagnostic process, a significant proportion (34.8%) of respondents held the belief that radiologists should have restricted control and instead rely on AI to provide recommendations. However, a fact worth noting is that 30% of the respondents held the belief that diagnostic decisions are evenly distributed between radiologists and AI systems (Fig. 2).

### Trust in AI

When asked about their level of trust in the decisions made by AI algorithms in radiography, 51.5% of the participants trusted AI decisions, 22.7% completely distrusted AI decisions, and 25.3% were neutral.

### Factors influencing trust

Participants were asked about the factors influencing their level of trust in AI-assisted radiography. Commonly cited factors include the accuracy of AI algorithms, transparency in AI decision-making processes, adherence to ethical guidelines for AI development, and personal experience with AI technologies.

### Preparedness for AI integration

Concerning their preparedness to adapt to the integration of AI in radiography, 65.8% of participants reported feeling prepared, 11.7% were neutral, and 22.5% reported feeling unprepared.

### Open-ended responses

**Fig. 3:**
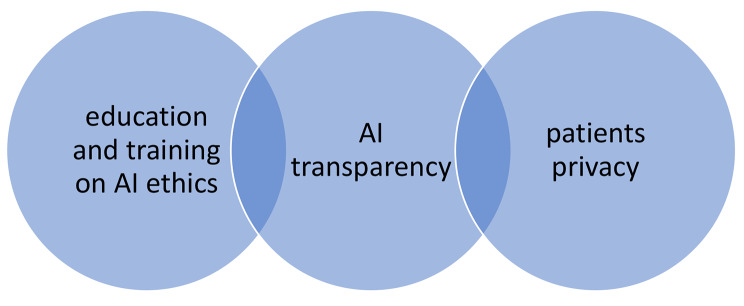
Themes regarding radiographers*’* perspectives on ethical considerations of artificial intelligence (AI) in radiography

The qualitative data obtained from the open-ended questions were subjected to thematic analysis. The key themes included concerns regarding patient privacy, the need for ongoing education and training in AI ethics, and the desire for increased transparency in AI algorithms. The dominant theme expressed by 217 respondents was the need for ongoing education and training in AI ethics. Representative quotes illustrating these themes are provided in Fig. 3.

## Discussion

The aim of this study was to explore radiographers’ perspectives on the ethical implications of AI in their field and identify their key concerns and potential strategies for addressing them. The results presented in this paper provide a comprehensive overview of radiographers’ perspectives on the ethical landscape of AI in radiography. These findings highlight the varying levels of familiarity, trust, and preparedness among radiographers, thereby emphasizing the importance of addressing ethical considerations in the integration of AI technologies in radiographic practice.

The integration of AI into radiography represents a transformative shift in medical imaging, offering unprecedented opportunities for improved diagnostics and workflow efficiency. However, AI technologies have become increasingly prevalent; therefore, ethical considerations surrounding their implementation have become paramount. This cross-sectional study examined the perspectives of radiographers, who are crucial stakeholders in the use of AI in medical imaging and sheds light on the ethical landscape and provided insights for the responsible development and implementation of these technologies.

### Understanding radiographers’ perspectives

The findings of this study revealed a nuanced understanding of AI among radiographers, reflecting varying degrees of familiarity and preparedness. Although some radiographers demonstrated a high level of awareness of and trust in AI, other radiographers were hesitant and expressed concern. These varying perspectives can be attributed to the differences in education, training, and exposure to AI technologies in professional environments. Similar studies [10, 11] reported comparable results, highlighting diverse perspectives among radiographers on the adoption of AI technologies. Some radiographers acknowledged the potential of AI to improve diagnostic accuracy and workflow efficiency. In contrast, others were apprehensive about possible job displacement and the need for further training.

Considerable debate exists regarding the potential impact of AI on the radiography sector [12]. A study by Hardy and Harvey [12] examined the potential impact of AI on the radiography profession by analyzing current workflow and identifying areas where AI automation such as protocol planning, image acquisition, and processing may be implemented. This study provided a comprehensive understanding of the practical use of AI in radiography. Furthermore, a study [9] conducted in Saudi Arabia examined radiographers’ perspectives on the use of AI in diagnostic imaging. This qualitative research focused on the specific challenges and concerns that radiographers face when incorporating AI into their profession. It also provided valuable recommendations for the development and advancement of radiography.

The integration of AI into the field of radiology has elicited considerable interest and concern within the radiographer community. The utilization of AI techniques has exhibited a remarkable ability to autonomously discern intricate patterns within imaging data, thereby facilitating the provision of quantitative evaluations pertaining to radiographic attributes [13].

The advent of AI in the field of radiology has elicited apprehension among radiographers, as highlighted by Abuzaid et al. [8]. Nevertheless, the lack of comprehensive data pertaining to the viewpoints of radiographers regarding the integration of AI within the realm of radiology is imperative to acknowledge, as expounded by Rainey et al. [14] in their recent study. Comprehending radiographers’ attitudes and perceptions regarding AI is of great importance because of their indispensable role in facilitating the effective integration of AI advancements within the field of radiology, as highlighted by Chen et al. [15]

As the frontline users of AI applications in radiography, radiographers have a pivotal role in shaping the ethical dimensions of radiography implementation. Their perspectives on patient privacy, data security, bias, and transparency offer valuable insights into the practical challenges faced during the integration of AI into daily practice.

### Ethical considerations in AI-assisted radiography

The ethical considerations highlighted in this study align with broader discussions in the literature. Patient privacy and confidentiality have emerged as significant concerns, echoing the findings of studies more broadly focusing on AI in healthcare [16]. Ethical considerations regarding the use of AI in radiography have a vital role in ensuring patient safety, respecting privacy, and promoting equitable healthcare delivery. The ethical and professional implications of incorporating AI into radiology have been carefully scrutinized. Currie et al. [3] argue that the ethical application of AI in radiology should prioritize patient well-being, minimize harm, and ensure an equitable distribution of benefits and risks among stakeholders. A thorough comprehension of the ethical guidelines and principles regulating AI in healthcare, specifically in radiography, is essential [17].

The ethical considerations related to the integration of AI into radiography are multifaceted and encompass a range of crucial aspects. These include the preservation of patient data privacy, ensuring the confidentiality of sensitive information, and addressing complex issues of data ownership. Furthermore, the development and utilization of AI in healthcare must be approached within a strong ethical framework to ensure that its implementation aligns with established ethical principles and guidelines [18]. A comprehensive examination of AI precision, ethical quandaries, and predispositions, along with the conceivable effects of disparity in discriminatory practices and legal liabilities that may arise from the integration of AI technology within the domain of radiography, is necessary [3, 19]. Moreover, ethical concerns associated with the design and deployment of AI in the healthcare domain is important to emphasize. This highlights the necessity of incorporating ethical considerations into the developmental process of AI and advocating the establishment of comprehensive frameworks to facilitate such integration [20, 21].

Insights from radiographers regarding the ethical implications of AI in radiology are vital for the effective implementation of AI advancements in radiological procedures. Understanding and addressing these ethical problems are crucial to ensure the conscientious and ethical use of AI in radiology. Continued evaluation of the issue is crucial as the understanding of the effects and capabilities of AI grows and is crucial to ensure that AI tools adhere to revised ethical regulations and guidelines [22].

Bias and fairness in AI algorithms have been recognized as critical issues in various domains. This study’s findings emphasize their relevance in the context of radiography. Radiographers recognize the potential for bias in algorithmic decision-making and express the need for ongoing efforts to address and mitigate these biases. The issue of bias in algorithmic decision-making in radiography is a crucial subject that has received significant attention in recent research. Algorithmic decision-making is believed to be driven by the concept that algorithms, unlike humans, make decisions without considering the specific attributes of the person being evaluated [23]. Lee [24] suggested that the perception of fairness and trust in algorithmic decisions is influenced by different traits associated with human and algorithmic decision makers. The possibility that algorithmic clinical predictions contribute to health disparities is a concern, which highlights the importance of assessing algorithmic bias and fairness in healthcare decision-making and prediction [25].

Cognitive and systemic factors in radiology influence the occurrence of diagnostic errors. Cognitive biases such as anchoring, framing, and premature closure have been identified as particularly prone to errors in the interpretation of radiological findings [26]. Moreover, an analysis conducted by Pot et al. [27] sheds light on the existence of fair and unfair biases within the machine learning and radiology domains. This finding underscores the significance of actively addressing biases existing in datasets and in algorithms. Furthermore, acknowledging the pivotal contribution of the ACR Data Science Institute (Reston, VA, USA) to promoting health equity in the field of radiology is crucial. This acknowledgment highlights the profound clinical implications associated with the inadvertent bias that may arise from the use of AI visualization algorithms in radiological practice, as discussed by Allen and Dreyer [28–30].

This study also underscores concerns related to the transparency and explainability of AI systems. Radiographers expressed unease with the “black box” nature of these algorithms, emphasizing the importance of understanding how AI reaches its conclusions. This lack of transparency can affect radiographers’ trust in AI systems, which aligns with the findings of studies focusing on trust in AI in healthcare.

### Implications for practice and policy

The insights gained from this study have practical implications for integrating artificial intelligence into radiography. Ethical guidelines and educational programs should be tailored to address the specific concerns and perspectives of radiographers. Transparent communication regarding the development, validation, and deployment of AI algorithms is essential for building trust among radiographers and for ensuring their active engagement and collaboration with AI technologies.

Moreover, this study’s findings underscored the need for ongoing professional development opportunities to enhance the preparedness of radiographers for the evolving AI landscape in radiography. As AI technologies continue to advance, continuous education and training programs will empower radiographers to effectively navigate ethical challenges and contribute to the responsible use of AI in healthcare.

### Limitations

This study acknowledges certain limitations, including the reliance on self-reported data, potential selection bias in the convenience sample, and inherent subjectivity associated with qualitative data analysis. These limitations are discussed in the interpretation of the results.

## Conclusions

This cross-sectional study provided valuable insights into the ethical considerations surrounding AI in radiography from the perspective of radiographers. By addressing their concerns and perspectives, an ethical framework can be fostered that promotes the responsible integration of AI, thereby ensuring that its benefits are realized while mitigating potential ethical pitfalls. AI adoption in the healthcare sector necessitates the crucial process of integrating technology and enhancing workforce skills. Continued discussion and updating of the guidelines are necessary to address the many ethical and legal challenges that arise at the algorithm, data, and clinical levels. This action will ensure that healthcare personnel have clear instructions to adhere to. Additional studies are necessary to comprehensively grasp the health–economic consequences and testing protocols necessary to guarantee that systems satisfy the specified performance standards while also preventing hidden biases.

Furthermore, defining the legal responsibilities of corporations and healthcare providers explicitly when utilizing such systems is imperative.

### Electronic supplementary material

Below is the link to the electronic supplementary material.

Supplementary Material 1

## Data Availability

Data are available upon request. Please Contact Dr. Faten Aldhafeeri, Email: fmaldhafeeri@uhb.edu.sa to obtain access to the raw data analyzed in this study.

## Abbreviations

AI: Artificial intelligence
