# Supplementary material for: Navigating the ethical landscape of artificial intelligence in radiography: a cross-sectional study of radiographers’ perspectives
Source: BMC Med Ethics. 2024 May 11;25:52. doi: 10.1186/s12910-024-01052-w (PMC11088142; doi:10.1186/s12910-024-01052-w)
Supplement: Supplementary file 1 — Supplementary Material 1 [file 12910_2024_1052_MOESM1_ESM.docx]

**Appendix 1**

**QUESTIONNAIRE**

**Navigating the Ethical Landscape of Artificial Intelligence in Radiography: A Cross-sectional Study of Radiographers’ Perspectives**

**Section A:** Participant Demographics

We will not collect any information that will lead to personal identification, however it would be very helpful to know your sex, where you work and general demographics.

1. **What is your sex?**
2. Male
3. Female
4. Prefer not to say *we should consider trans gender….*
5. **What is your age?**
6. 22 - 32 years
7. 33 - 43 years old
8. 44 - 53 years old
9. >54
10. **Which best describes the organisational set-up or structure of your facility?**
11. Government Facility
12. Quasi-government Facility
13. Military Facility
14. Private Facility
15. Other
16. **How long have you been working as a radiographer?**
17. <5 years
18. 6-10 years
19. 11 - 15 years
20. 16 - 20 years

e. 21 - 25 years

f. >26 years

1. **What is your highest level of education (academic qualification attained)?**
2. Certificate
3. Diploma
4. Bachelor’s degree
5. Master’s degree
6. PhD
7. Others
8. **Which medical imaging modality (s) do you use for your daily work or are you competent at using for your assigned roles? (***Please select those that apply***)**
9. General X-ray
10. Computed Tomography
11. Magnetic Resonance Imaging
12. Fluoroscopy
13. Mammography
14. Ultrasound
15. Dental X-ray
16. Others
17. **How familiar are you with the concept of artificial intelligence in radiology?**
18. Very familiar
19. Familiar
20. Neutral
21. Not familiar
22. Not familiar at all

**Section B:** General Attitudes and Perspectives

Please let us know about any general attitudes you have about the emerging integration and use of AI in medical imaging in Saudi Arabia.

1. **I am aware of AI as an emerging trend in medical imaging in Saudi Arabia.**
2. Strongly Agree
3. Agree
4. Not Sure
5. Disagree
6. Strongly Disagree
7. **AI technology would improve general radiography practice and quality assurance for its efficient diagnosis and improved clinical care of my patients.**
8. Strongly Agree
9. Agree
10. Not Sure
11. Disagree
12. Strongly Disagree
13. **The integration of AI into medical imaging practice in Africa would introduce more benefits than harm.**

**a.** Strongly Agree

b. Agree

c. Not Sure

d. Disagree

e. Strongly Disagree

1. **Have you received any training on the use of artificial intelligence tools in radiology?**
2. Yes
3. No

**Section C: impact of ethical considerations on radiographers’ practices**

Could you kindly provide your viewpoint on the ethical implications that arise from the growing integration and utilisation of artificial intelligence in the field of medical imaging in Saudi Arabia?

1. **How concerned are you about patient privacy when utilizing artificial intelligence in radiology**?
2. Very concerned
3. Somewhat concerned.
4. Neither concerned nor unconcerned
5. Somewhat unconcerned
6. Very unconcerned
7. **Do you think there should be specific ethical guidelines for the use of artificial intelligence in radiology?**
8. Yes
9. Maybe
10. No
11. **To what extent do you believe radiologists should have control over AI-driven decisions in the diagnostic process?**
12. Complete control
13. Significant input with AI assistance
14. Equal partnership between radiologist and AI
15. Limited control with AI providing recommendations.
16. Full autonomy for AI-driven decision
17. **How important is it for AI systems in radiology to be transparent and provide explanations for their decisions?**
18. Extremely important
19. Somewhat important
20. Neutral
21. Somewhat not important
22. Extremely not important
23. **How do you think the implementation of AI in radiology should be regulated to ensure ethical practices? (Open-ended)**
